# Prediction value with a novel and accurate tissue‐based human papillomavirus detection method in low‐grade squamous intraepithelial lesions

**DOI:** 10.1002/cam4.4634

**Published:** 2022-03-28

**Authors:** He Wang, Zheng He, Xue Han, Deyu Zhang, Shitai Zhang

**Affiliations:** ^1^ Department of Obstetrics First Affiliated Hospital of China Medical University Shenyang China; ^2^ Department of Obstetrics and Gynecology Shengjing Hospital of China Medical University Shenyang China

**Keywords:** human papillomavirus, low‐grade squamous intraepithelial lesions, uniplex E6/E7 PCR

## Abstract

**Background:**

The progression rate from CIN1 to CIN3 is 9.0% and that for invasive cancer is 1.0%. The large majority of CIN1 lesions regress spontaneously, and the treatment of CIN1 is still controversial.

**Aims:**

The aim of this study is to investigate the responsible HPV genotype in the low‐grade SILs, then to predict the presence of high‐grade SILs, and determine whether further treatment is needed.

**Methods:**

We use the methods of manual microdissection with FFPE tissue specimens and the E6/E7 uniplex polymerase chain reaction (PCR) to detect HPV in the lesions.

**Results:**

The HPV test was performed on 72 biopsy tissue specimens, and 55 (76.4%, 55/72) of them were HPV positive. Nine (16.4%, 9/55) of them escalated to CIN2 after LEEP or cervical conization, and 46 (83.6%, 46/55) were still CIN1. There were 17 (23.6%, 17/72) cases with HPV‐negative results in cervical biopsy tissues.

HPV test of cervical biopsy diagnosed with CIN1 has a positive predictive value of 16.4% in the presence of CIN2 or higher lesions, a negative predictive value of 94.1%, a specificity of 25.8%, and a sensitivity of 90.0%. HPV test of cervical biopsy tissues for the prediction of HPV infection in LEEP or cone surgery tissues had a positive predictive value of 80.0%, a negative predictive value of 82.3%, a specificity of 56.0%, and a sensitivity of 93.6%.

**Conclusions:**

It is the first time that we have detected HPV genotype in the low‐grade SILs by the methods of manual microdissection with FFPE tissue specimens and the E6/E7 uniplex PCR. Patients with cervical biopsy tissue diagnosed with CIN1 and with a negative or only low‐risk HPV type result can be considered for follow‐up. Conversely, in cases of cervical biopsy tissue diagnosed with CIN1 positive for high‐risk HPV, surgery or a close follow‐up program can be selected.

## Abstract 2

Patients with cervical biopsy tissue diagnosed with CIN1 and with a negative or only low‐risk HPV type result can be considered only follow‐up. Conversely, in cases of cervical biopsy tissue diagnosed with CIN1 positive for high‐risk HPV, surgery or a close follow‐up program can be selected.

## 1 INTRODUCTION

Cervical cancer is the second most common malignant tumor in women worldwide.^1^ It is well established that most cervical cancers develop from noninvasive dysplastic lesions known as cervical intraepithelial neoplasia (CIN).^2^, ^3^ There are three categories of CINs based on the degree of dysplasia, namely CIN1 (mild), CIN2 (moderate), and CIN3 (severe).^4^ Early detection and diagnosis of these precancerous lesions, which were recently called squamous intraepithelial lesions (SILs),^5^ are essential to prevent cervical cancer.

Since Strauss discovered the human papillomavirus (HPV) under the electron microscope in 1949, Zur Hausen first proposed that HPV is closely related to cervical cancer in 1974.^6^ This virus is a kind of papillomavirus and belongs to the small, nonenveloped, and double‐strand DNA virus type. After decades of research, HPV is currently thought to be a causative agent of cervical cancer and its precursors.^7^, ^8^ Until now, about 200 types of HPV have been identified, and nearly 25% of them have been detected in the mucosal epithelium of the female genital tract.^9^, ^10^ Based on many epidemiological and clinical studies, the World Health Organization (WHO) classifies mucosal HPV into 12 high‐risk (HR) HPV types (HPV16, 18, 31, 33, 35, 39, 45, 51, 52, 56, 58, and 59) and a probable high‐risk type (HPV68).^11^ Additionally, nine HPV types (HPV26, 30, 34, 53, 66, 67, 70, 73, and 82) are thought to be possible high‐risk HPV (pHR) types, as they are occasionally identified in malignant tumor tissue.^11^, ^12^, ^13^ HR‐HPV encodes two oncoproteins, E6 and E7, which are continuously transcribed after the integration of the HPV genome into a host cell’s DNA.^11^ The E6 and E7 are synergistically acted to drive the host cells to proliferate and transform those into cancer cells. It is also known that such actions are generally not present in low‐risk (LR) HPV types.

Cervical HPV infection is the most common sexually transmitted disease. However, about 90% of them are transient, and these viruses can be cleared from the patient within a few years.^14^, ^15^ Nevertheless, some patients have persistent infections and develop high‐grade SILs (CIN2 and CIN3) several years later or even cervical cancer more than 10–20 years later.^15^, ^16^, ^17^

Epidemiological studies have shown that HPV in cervical squamous cell carcinoma (Cx‐SCC) has a prevalence of 98.7%,^15^ while CIN2 and CIN3 have a prevalence of 83.6% and 93.2%, respectively. Contrastingly, in CIN1 (low‐grade SIL) patients, the HPV prevalence is considerably lower (in the range of 30.6%–74.8%).^15^, ^18^ We have previously reported that in cervical cancer and high‐grade SIL lesions, multiple HPV types are frequently identified in the scraped cervical cell samples, but finally, only one HPV type is found in one tissue lesion.^14^, ^19^ This suggests that cell‐sample is not suitable to determine HPV type responsible for the target lesion, a combination of microdissection from FFPE (formalin‐fixed, paraffin‐embedded) tissue specimen and the highly sensitive HPV test may be the most accurate method to determine the relationship between responsible HPV type and the related lesion.^20^

The progression rate from CIN1 to CIN3 is 9.0% and that to invasive cancer is only 1.0%. In fact, the large majority of CIN1 lesions (57%–90%) regress spontaneously,^13^ thus, the overtreatment of CIN1 should be minimized while avoiding the missed diagnosis of high‐grade SILs and even cervical cancer, which is particularly meaningful for women seeking to have children, since the excisional procedures may result in adverse obstetric outcomes in the future. At present, there is a lack of consensus regarding the medical intervention for CIN2 and CIN3 lesions, which should be treated by excision of the transformation zone with a postoperative follow‐up of at least 2–5 years to observe for possible recurrence, or less commonly, hysterectomy for CIN3 or carcinoma in situ,^21^, ^22^ however, the treatment of CIN1 is still controversial.^23^, ^24^ The Japanese Treatment Guideline recommends that CIN1 should be monitored rather than treated because 70% of CIN1 cases spontaneously regress within 1 year and 90% regress within 2 years.^25^

Therefore, the main target of a predictive test is to have an extremely high or even an absolute negative predictive value, which means that no cases were predicted to be low‐grade SILs. However, the finding of high‐grade SIL had an acceptable positive predictive value. With such a predictive test, it may be possible to reduce the number of patients overtreated for CIN1 lesions that may not progress or may even regress spontaneously.

The aim of this study is to investigate the detection of HPV genotype responsible for the lesion by the methods of manual microdissection with FFPE tissue specimens and the E6/E7 uniplex polymerase chain reaction (PCR), then to predict the presence of higher‐grade SIL lesions, and determine whether further treatment is needed.

## 2 MATERIALS AND METHODS

### 2.1 Selection of subjects and specimens

We selected patients who underwent cervical cancer screening at the Gynecological Clinic of China Medical University Affiliated Shengjing Hospital from January 2017 to January 2019. Due to abnormal results in the cytological test (Thinprep Cytologic Test) and HPV test using cervical canal cells, 5367 patients underwent further colposcopy or cervical multiple‐point biopsy in the Cervical Lesion Clinic. The endpoint diagnosis was based on the histopathological diagnosis, and the highest grade was chosen (i.e., if four biopsy tissue specimens from one case were diagnosed as CIN1, CIN2, CIN1, and chronic cervicitis, CIN2 was determined as the endpoint diagnosis). In total, 1033 patients were diagnosed as CIN1, and only 76 of them underwent further treatment, including the Loop Electrosurgical Excision Procedure (LEEP) or cervical diagnostic conization, while the remaining 957 cases were simply monitored with a cytological test, HPV test, and colposcopy. Consent was obtained from all subjects before surgery for the use of their cells or histological specimens for this study, and the protocol was approved by the Ethics Committee of China Medical University Shengjing Hospital.

The Thinprep Cytologic Test (TCT) was performed through a liquid‐based thin layer machine (ThinPrep, Hologic) and was then examined and diagnosed by an experienced cytopathologist. The criteria of diagnosis were based on The Bethesda System classification revised in 2001.^4^ Abnormal results included atypical squamous epithelial cells with unclear meaning (ASCUS), low‐grade squamous intraepithelial lesions (LSIL), atypical squamous epithelial cells that do not exclude high‐grade squamous intraepithelial lesions (ASC‐H), and high‐grade squamous intraepithelial lesions (HSIL). On the other hand, the absence of intraepithelial lesions or malignant cells was considered a normal result.

The HPV test of cervical canal cells was performed using the HPV typing test kit (Kaipu Bio Ltd). The kit can identify 13 high‐risk types (HPV16, 18, 31, 33, 35, 39, 45, 51, 52, 56, 58, 59, and 68), two possible high‐risk types (HPV53 and HPV66), and six low‐risk types (HPV6, 11, 42, 43, 44, and 81). A positive result for more than one type was considered a multi‐type infection. However, five possible high‐risk types, namely HPV26, 67, 70, 73, and 82, could not be detected. The TCT and HPV tests were performed within 1 week from the first visit.

Colposcopy was performed to examine the presence of cervical lesions and then three or four punch biopsy specimens were obtained from abnormal lesions. Both the steps were performed by an experienced gynecologist.

As for further treatment, LEEP surgery was performed in the Cervical Lesion Clinic, whereas cervical conization was completed in the operating room. All final histopathological diagnoses were revised in consensus by two experienced pathologists. If the diagnosis results were inconsistent, a third high‐level pathologist assisted the diagnosis.

### 2.2 Sandwich cutting for FFPE tissue specimens and DNA extraction

With the assistance of a pathologist, we obtained all the 72 patients’ FFPE blocks, which contained biopsy tissue and LEEP or conization tissue.

The FFPE blocks were sectioned according to the following procedure: 1 slide of 4 μm section was used for histopathological diagnosis, and 4–5 10 μm slides were used for DNA extraction and HPV genotyping, and the last 4 μm slide was used for histopathological evaluation to confirm target lesion remained. The microtome was cleaned with 70% alcohol, and the blade was replaced with each tissue block in order to avoid contamination. The tissue slides were stored in a refrigerator at −20°C until the next procedure.

All the control slides with HE staining were reviewed by the same pathologist, and the final diagnoses were confirmed. The areas of abnormal lesions, and normal squamous or glandular epithelium were marked separately on each control slide. Viewing the marked areas, abnormal area, or normal epithelium on the tissue slides were independently dissected by hand using a sterilized needle under the inverted microscope. Each tissue fragment was placed into a 1.5 ml tube with 50 μl of alkaline lysis reagent (25 mM NaOH, 0.2 mM EDTA, pH 12.0). Next, the tube was incubated in a thermo‐shaker (Biosan, TS‐100, Latvia) at 300 rounds per min (rpm) for 15 min at 95°C, the tube was then spun down for 10 s, and the procedure was repeated at 300 rpm once, at 900 rpm twice. Subsequently, the same amount of acidic neutralizing solution (0.04 mM Tris–HCL, pH 5.0) was added into the tube, mixed thoroughly, and centrifuged (12,000 rpm) for 1 min. Finally, 30–50 μl of supernatant was transferred into a new 1.5 ml tube and diluted 5 times with distilled water (DW), and 5 μl of the solution was used in the next procedure for HPV genotyping, and the remaining solution was stored in a freezer at −20°C.

### 2.3 HPV genotyping using uniplex E6/E7 PCR methods

The uniplex E6/E7 PCR test was designed to amplify E6 and E7 genes of 39 common HPV types using type‐specific primer pairs. This assay was able to detect 13 high‐risk types (HPV16, 18, 31, 33, 35, 39, 45, 51, 52, 56, 58, 59, and 68), 11 possible high‐risk types (HPV26, 30, 34, 53, 66, 67, 69, 70, 73, 82, and 85), 15 low‐risk types (HPV6, 11, 40, 42, 44, 54, 55, 61, 62, 71, 74, 81, 84, 89, and 90), and the beta‐globin gene as a positive control individually. The specific procedures have been described in previous papers.^26^ Cases that were negative for the beta‐globin gene (possibly due to poor quality of the sample or insufficient amount of extracted DNA) were excluded from further analysis.

### 2.4 Statistical analysis

We used the Mann–Whitney *U* test to analyze continuous variables (patient’s age), and the Chi‐square test with Yates’ correction was used to compare categorical variables (prevalence of HPV or cytological result). Fisher’s exact test was adopted when the total sample size was less than 10. A cutoff value of *p* < 0.05 was considered statistically significant. All the statistical analyses were performed with SPSS version 19.0 (IBM).

## 3 RESULTS

### 3.1 Overall cytological diagnosis and HPV prevalence in all subjects

**FIGURE 1:**
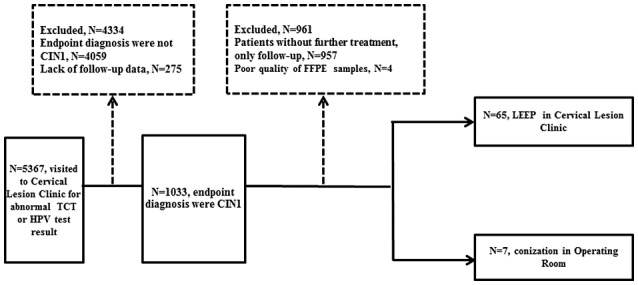
Stratification of patients investigated for low‐grade cervical squamous intraepithelial patients

A total of 72 patients were included with an average age of 41.8 (23–62 years) years. The prevalence of ASCUS+, LSIL+, and HSIL (or ASC‐H) among them was 100.0%, 85.3%, and 25.0%, respectively (Table 1). Among them, HR‐HPV in cervical canal cells was detected in 71 patients, and one patient had a negative cytological HPV result. All the 72 patients had a colposcopy biopsy and were diagnosed with CIN1. The HPV test was performed in all 72 biopsy tissue specimens, and 55 (76.4%, 55/72) of them were HPV positive, including 25 cases of HR‐HPV types and 2 cases of pHR‐HPV types, whereas the other 28 were LR‐HPV types (Table 1). Following the cervical biopsy, 65 patients underwent LEEP in the Cervical Lesion Clinic and 7 patients underwent cervical conization in the operating room (Figure 1).

**TABLE 1:** Overall cytological diagnosis and HPV prevalence in biopsy and LEEP (or conization) groups

	Biopsy group	LEEP (or conization) group		
Diagnosis	CIN1	CIN1	CIN2	*p* value^a^
Age	41.8 (23–62 years)	40.4 (23–61 years)	50.3 (34–62 years)	0.005
ASCUS+	72 (100)	62 (100)	10 (100)	
LSIL+	60 (83.3)	52 (83.9)	8 (80.0)	0.879
ASC‐H or HSIL	18 (25.0)	12 (19.4)	6 (60.0)	0.018
HPV prevalence (%)	55 (76.4)	37 (59.7)	10 (100)	0.095
HR‐HPV (%)	25 (34.7)	19 (30.6)	10 (100)	<0.001
pHR‐HPV (%)	2 (2.8)	2 (3.2)	0	0.645
LR‐HPV (%)	28 (38.9)	27 (43.5)	0	0.022
Negative (%)	17 (23.6)	14 (22.6)	0	0.214
Total	72	62	10	

Abbreviations: ASCUS, atypical squamous epithelial cells with unclear meaning; ASC‐H, atypical squamous epithelial cells do not exclude high‐grade squamous intraepithelial lesions; CIN, cervical intraepithelial neoplasia; HPV, human papillomavirus; HSIL, high‐grade squamous intraepithelial lesions; HR, high risk; LEEP, loop electrosurgical excision procedure; LR, low risk; LSIL, low‐grade squamous intraepithelial lesions; pHR, possible high risk.

^a^ *p* value means the difference between CIN1 and CIN2 patients in the LEEP (or conization) group; ASCUS+, worse than ASCUS; LSIL+, worse than ASCUS.

There were 11 cases with HPV‐positive result in biopsy tissue but HPV‐negative result in postoperative tissue specimens (details in Table 4). This may have been caused by the destruction of the lesions through the biopsy and postoperative scorch hemostasis. We included these cases in the HPV‐positive group.

Finally, there were 10 (13.9%, 10/72) patients with postoperative pathological escalation to CIN2, and 62 (86.1%, 62/72) patients were still CIN1. No escalation to CIN3 or worse was found (Table 1). The average age of the 10 pathological upgrade patients was 50.3 years, higher than that of the patients without the pathological upgrade group (40.4 years, *p* = 0.005). The prevalence of HR‐HPV, pHR‐HPV, and LR‐HPV in the unescalated group was 30.6%, 3.2%, and 43.5%, respectively, and that for the escalated group was 100.0%, 0, and 0, respectively (Table 1). HR‐HPV was more common in the escalated group than in the unescalated group (*p* < 0.001), whereas LR‐HPV type was more often in the unescalated group (*p* < 0.022). Of the 72 LEEP (or conization) issue specimens, 14 were negative for HPV, and all of them were CIN1 (Table 1).

### 3.2 The prediction value of HPV results in the cervical biopsy tissue

Of the 71 HPV‐positive patients with cervical canal cells, 56 patients were still HPV positive (78.9%, 56/71) in tissue specimens after cervical biopsy or LEEP (or conization), and 15 patients (21.1%, 15/71) were negative both in cervical biopsy and LEEP (or conization) tissue specimens.

Among the 72 cervical biopsy tissues, 55 (76.4%, 55/72) cases were positive with HPV, 9 (16.4%, 9/55) of them escalated to CIN2 after LEEP or cervical conization, and 46 (83.6%, 46/55) were still CIN1 (Table 2). There were 17 (23.6%, 17/72) cases with HPV‐negative results in cervical biopsy tissues, including one (5.9%, 1/17) case that upgraded to CIN2 after LEEP or cervical conization, with the remaining 16 (94.1%, 16/17) cases having unchanged pathological diagnosis (Table 2). No pathological diagnosis of CIN3 or worse was found in either group. It seemed that the risk of postoperative pathological escalation in patients with HPV‐positive results in cervical biopsy tissue (16.4%, 9/55) was higher than that in patients with HPV‐negative results (5.9%, 1/17), however, the difference was not statistically significant (*p* = 0.49). At the same time, it can be concluded that the HPV test of cervical biopsy diagnosed with CIN1 has a positive predictive value of 16.4% for the presence of CIN2 or higher lesions, a negative predictive value of 94.1%, a specificity of 25.8%, and a sensitivity of 90.0%.

**TABLE 2:** HPV result with cervical biopsy tissue in prediction of LEEP (or conization) tissue pathological diagnosis

	LEEP (or conization) pathological diagnosis			
	CIN1	CIN2	Total	*p* value
HPV (+) with cervical biopsy tissue	46	9	55	0.49
HPV (−) with cervical biopsy tissue	16	1	17	
Total	62	10	72	

Abbreviations: CIN, cervical intraepithelial neoplasia; HPV, human papillomavirus; LEEP, loop electrosurgical excision procedure.

Of the 55 patients with HPV‐positive results in cervical biopsy tissue, 44 patients (80.0%, 44/55) were still HPV positive in LEEP (or conization) tissue specimens, however, 11 cases were HPV negative (20.0%, 11/55) after LEEP (or conization) (Table 3). Of the 17 HPV‐negative patients with cervical biopsy, 14 (82.3%, 14/17) were still negative in LEEP (or conization) tissue specimens, and two patients (17.6%, 3/17) had positive HPV after LEEP (or conization) (Table 3). The HPV test of cervical biopsy tissues for the prediction of HPV infection in LEEP or cone surgery tissues had a positive predictive value of 80.0%, a negative predictive value of 82.3%, a specificity of 56.0%, and a sensitivity of 93.6%.

**TABLE 3:** HPV result with cervical biopsy tissue in the prediction of HPV result with LEEP (or conization) tissue

	HPV result with LEEP (or conization) tissue		
	(+)	(−)	Total
HPV (+) with cervical biopsy tissue	44	11	55
HPV (−) with cervical biopsy tissue	3	14	17
Total	47	25	72

Abbreviations: HPV, human papillomavirus; LEEP, loop electrosurgical excision procedure.

### 3.3 HPV genotype in LEEP (or conization) pathological diagnosis unchanged and upgrade groups

Among the 72 tissues, after cervical LEEP or conization, there were 10 cases of CIN2, and high‐risk HPV was detected in all of them; 9 cases were single‐type infections, of which HPV16 was detected in 4 cases, HPV52 in 2 cases, and HPV18/51/58 only in 1 case for each type (Table 4), and there was one case of multiple‐type infection with HPV51 and 42 (case no. 68).

**TABLE 4:** Cytological diagnosis and HPV type in LEEP (or conization) pathological diagnosis upgrade group

Case no.	Age	Cytological diagnosis	HPV type in cervical canal cells	Biopsy diagnosis	HPV type in biopsy tissue	LEEP (or conization) diagnosis	HPV type in LEEP (or conization) tissue	Ultimate HPV type in tissue^a^
19	41	LSIL	16	CIN1	16	CIN2	16	16
34	47	ASCUS	52	CIN1	52	CIN2	52	52
49	61	ASCUS	18, 58	CIN1	18	CIN2	18	18
51	56	HSIL	16, 51	CIN1	16	CIN2	16	16
56	49	HSIL	16, 51	CIN1	51	CIN2	51	51
61	59	LSIL	16	CIN1	16	CIN2	16	16
65	52	HSIL	16, 31	CIN1	16	CIN2	16	16
66	34	HSIL	52	CIN1	52	CIN2	52	52
68	57	HSIL	51	CIN1	51, 42	CIN2	51, 42	51, 42
71	47	HSIL	58	CIN1	Negative	CIN2	58	58

Abbreviations: ASCUS, atypical squamous epithelial cells with unclear meaning; CIN, cervical intraepithelial neoplasia; HPV, human papillomavirus; HSIL, high‐grade squamous intraepithelial lesions; LEEP, loop electrosurgical excision procedure; LR, low risk; LSIL, low‐grade squamous intraepithelial lesions.

^a^ Ultimate HPV type in tissue means combining the HPV type in biopsy and LEEP (or conization).

There were still 62 cases of CIN1 after surgery, of which 14 (22.6%, 14/62) cases were negative. Among 48 (77.4%, 48/62) cases that were positive for HPV, 6 were positive with HPV58, 3 were positive with HPV16/51/52/68, and 2 were positive with HPV35/39/45/53. The least frequent were HPV18, 31, 56, and 59, with only one case for each type, and there were 27 cases of low‐risk HPV infection (Table 5).

**TABLE 5:** Cytological diagnosis and HPV type in LEEP (or conization) pathological diagnosis unchanged group

Case no.	Age	Cytological diagnosis	HPV type in cervical canal cells	Biopsy diagnosis	HPV type in biopsy tissue	LEEP (or conization) diagnosis	HPV type in LEEP (or conization) tissue	Ultimate HPV type in tissue^a^
1	23	LSIL	39, 51	CIN1	LR‐HPV (54)	CIN1	LR‐HPV (54)	LR‐HPV (54)
2	23	LSIL	39	CIN1	Negative	CIN1	Negative	Negative
3	24	LSIL	39, 51	CIN1	LR‐HPV (90)	CIN1	LR‐HPV (90)	LR‐HPV (90)
4	25	LSIL	39	CIN1	Negative	CIN1	Negative	Negative
5	26	LSIL	33	CIN1	LR‐HPV (90)	CIN1	LR‐HPV (71, 90)	LR‐HPV (71, 90)
6	26	ASC‐H	16, 58, 44	CIN1	Negative	CIN1	16, 58	16, 58
7	27	HSIL	16, 58	CIN1	Negative	CIN1	58	58
8	28	LSIL	18, 52	CIN1	Negative	CIN1	Negative	Negative
9	28	LSIL	16, 52	CIN1	LR‐HPV (55)	CIN1	LR‐HPV (55)	LR‐HPV (55)
10	29	LSIL	18, 52	CIN1	LR‐HPV (74)	CIN1	LR‐HPV (74)	LR‐HPV (74)
11	30	LSIL	35, 51	CIN1	35, 51	CIN1	35, 51	35, 51
12	31	LSIL	35, 51	CIN1	LR‐HPV (54)	CIN1	LR‐HPV (54)	LR‐HPV (54)
13	32	LSIL	16	CIN1	16, 52	CIN1	16	16, 52
14	32	HSIL	16	CIN1	LR‐HPV (71)	CIN1	LR‐HPV (71)	LR‐HPV (71)
15	32	HSIL	16, 58	CIN1	Negative	CIN1	Negative	Negative
16	33	LSIL	45	CIN1	45	CIN1	Negative	45
17	33	LSIL	16, 52	CIN1	16, 52	CIN1	16, 52	16, 52
18	34	LSIL	52	CIN1	Negative	CIN1	Negative	Negative
20	35	LSIL	16	CIN1	Negative	CIN1	Negative	Negative
21	36	LSIL	35	CIN1	35	CIN1	35	35
22	36	LSIL	16, 52	CIN1	LR‐HPV (90)	CIN1	LR‐HPV (90)	LR‐HPV (90)
23	37	LSIL	39	CIN1	39	CIN1	39	39
24	38	LSIL	58	CIN1	59	CIN1	Negative	59
25	38	HSIL	18	CIN1	LR‐HPV (42)	CIN1	LR‐HPV (42)	LR‐HPV (42)
26	39	LSIL	58, 68	CIN1	58, 68	CIN1	Negative	58, 68
27	39	HSIL	18	CIN1	Negative	CIN1	Negative	Negative
28	39	LSIL	31, 35	CIN1	LR‐HPV (84)	CIN1	LR‐HPV (71, 84)	LR‐HPV (71, 84)
29	39	LSIL	31, 35	CIN1	Negative	CIN1	Negative	Negative
30	40	LSIL	51, 58	CIN1	LR‐HPV (54)	CIN1	LR‐HPV (54)	LR‐HPV (54)
31	40	LSIL	16, 51, 52	CIN1	LR‐HPV (55)	CIN1	LR‐HPV (55)	LR‐HPV (55)
32	40	ASCUS	51, 58	CIN1	Negative	CIN1	Negative	Negative
33	40	ASCUS	51, 58	CIN1	LR‐HPV (62)	CIN1	LR‐HPV (62)	LR‐HPV (62)
35	41	LSIL	56	CIN1	56	CIN1	56	56
36	41	LSIL	58, 68	CIN1	LR‐HPV (84)	CIN1	Negative	LR‐HPV (84)
37	42	LSIL	35	CIN1	LR‐HPV (71)	CIN1	LR‐HPV (71)	LR‐HPV (71)
38	42	LSIL	39	CIN1	45	CIN1	Negative	45
39	43	LSIL	68	CIN1	68	CIN1	Negative	68
40	43	LSIL	68	CIN1	68	CIN1	68	68
41	44	ASCUS	56	CIN1	31, 39	CIN1	31, 39	31, 39
42	45	LSIL	59	CIN1	LR‐HPV (90)	CIN1	LR‐HPV (90)	LR‐HPV (90)
43	45	ASCUS	52, 58	CIN1	LR‐HPV (55)	CIN1	Negative	LR‐HPV (55)
44	45	ASCUS	51, 59	CIN1	Negative	CIN1	Negative	Negative
45	46	ASCUS	52, 58	CIN1	52,58	CIN1	52, 58	52, 58
46	46	LSIL	52	CIN1	LR‐HPV (84,90)	CIN1	LR‐HPV (84)	LR‐HPV (84, 90)
47	46	LSIL	negative	CIN1	Negative	CIN1	Negative	Negative
48	46	LSIL	56	CIN1	LR‐HPV (40)	CIN1	LR‐HPV (40)	LR‐HPV (40)
50	47	HSIL	31, 39	CIN1	LR‐HPV (74)	CIN1	Negative	LR‐HPV (74)
52	47	ASCUS	51, 59	CIN1	Negative	CIN1	Negative	Negative
53	48	LSIL	18, 51	CIN1	18, 51	CIN1	Negative	18, 51
54	48	HSIL	52, 68	CIN1	LR‐HPV (40)	CIN1	LR‐HPV (40)	LR‐HPV (40)
55	48	HSIL	52, 68	CIN1	Negative	CIN1	Negative	Negative
57	49	ASCUS	68	CIN1	LR‐HPV (62)	CIN1	LR‐HPV (62)	LR‐HPV (62)
58	50	ASC‐H	31, 39	CIN1	LR‐HPV (84)	CIN1	LR‐HPV (84)	LR‐HPV (84)
59	51	LSIL	39, 53	CIN1	53	CIN1	53	53
60	51	LSIL	16	CIN1	LR‐HPV (90)	CIN1	LR‐HPV (90)	LR‐HPV (90)
62	53	LSIL	16	CIN1	LR‐HPV (40)	CIN1	LR‐HPV (40)	LR‐HPV (40)
63	55	ASCUS	58	CIN1	58	CIN1	58	58
64	55	LSIL	18, 51	CIN1	LR‐HPV (55)	CIN1	Negative	LR‐HPV (55)
67	57	ASCUS	51, 56	CIN1	51, 58	CIN1	Negative	51, 58
69	59	LSIL	16	CIN1	Negative	CIN1	Negative	Negative
70	60	HSIL	53, 42	CIN1	53	CIN1	53	53
72	61	ASC‐H	52, 42	CIN1	LR‐HPV (42)	CIN1	LR‐HPV (42)	LR‐HPV (42)

Abbreviations: ASCUS, atypical squamous epithelial cells with unclear meaning; ASC‐H, atypical squamous epithelial cells do not exclude high‐grade squamous intraepithelial lesions; CIN, cervical intraepithelial neoplasia; HPV, human papillomavirus; HSIL, high‐grade squamous intraepithelial lesions; LEEP, loop electrosurgical excision procedure; LR, low risk; LSIL, low‐grade squamous intraepithelial lesions.

^a^ Ultimate HPV type in tissue means combining the HPV type in biopsy and LEEP (or conization).

## 4 DISCUSSION

Previous studies on how to avoid overlooking the diagnosis of CIN2 or worse have mainly focused on the analysis of related factors, such as patient age, pap smear test, and colposcopy results. However, this study is the first to use a highly sensitive uniplex PCR‐based HPV genotyping method in the biopsy tissue of the cervix to predict worse lesions.

Cervical cancer is one of the few malignant tumors with a clear etiology, and its high‐risk factors mainly include smoking, alcoholism, premature sex, and promiscuity.^27^ However, persistent HPV infection is the most important cause.^15^, ^27^ Because of the recent advances in research and therapy, people have realized that most HPV virus infections are self‐limiting and transient infections. The virus can be eliminated by the body within 1 or 2 years, and the virus will not be cleared from the body only in a small proportion of patients (about 4%), causing the infection to persist and leading to extended disease duration or further progression of cervical cancer.^14^, ^15^ Previous studies have shown that HPV infection is a necessary cause for high‐grade squamous intraepithelial lesions and even cervical cancer.^7^, ^28^ Meanwhile, with the commercial application of various HPV detection methods, HPV testing is considered to be equivalent or even superior to the cytological test in some European and American countries and has indeed become the preferred method of cervical cancer screening.^13^, ^29^

CIN has the characteristics of the multicentric distribution of lesions and coexistence of various degrees of lesions, and about 49.0% of CIN2 and higher‐grade lesions had invasive growth, and the surface epithelium had a completely normal appearance.^30^ Notably, even experienced gynecologic oncologists cannot fully detect all the lesions. According to previous reports, in the cases diagnosed as CIN1 under colposcopy biopsy, the missed diagnosis rate of CIN2 and higher‐grade lesions is 19%–55%.^31^ Van Delft et al. showed that among 109 CIN1 patients who underwent colposcopy, 47.71% were combined with CIN2 or worse.^32^ In addition, Kinney et al. and Barut et al. reported that CIN3 is often missed or misdiagnosed as CIN1 or CIN2 using cytology and colposcopy in the initial screening procedure, because of low sensitivity and specificity, or sometimes due to poor technique, incorrect biopsy site, or the presence of multiple lesions with different grades of CIN.^33^, ^34^ Thus, for patients with the endpoint diagnosis of CIN1, how to avoid missing higher‐grade lesions while avoiding overtreatment has become a crucial challenge among gynecologists. For the first time, we used the novel uniplex E6/E7 PCR method to determine the HPV prevalence and genotype using cervical biopsy FFPE tissues and to predict the presence of higher‐grade SILs and determine the following treatment method. Compared with the Cobas 4800 HPV test and Linear Array HPV genotyping test, which are approved by Food and Drug Administration, the E6/E7 method has the following advantages: first, the other two methods target the L1 region, whereas this assay targeting the E6/E7 genes may be more adequate for screening and prediction because the E6 and E7 genes are always preserved in cancer and precancerous tissues.^35^, ^36^ Second, we used type‐specific primers in the uniplex assay (one HPV type in one tube) and confirmed that this method has a high sensitivity, detecting as little as 100 viral copies without any cross‐reactivity, which indicates that it can minimize the occurrence of false negatives.^26^

In our study, 10 patients were finally diagnosed as CIN2 after LEEP or cervical conization. This means that higher‐grade lesions were missing during cervical biopsy under colposcopy in 13.89% of the patients, which was lower than the result of other studies.^31^, ^33^ The mean age of the 10 pathological upgrade patients was 50.3 years, which was higher than that of the patients without the upgrade (*p* = 0.005). This is consistent with the conclusions from previous studies, and it also shows that the progression of CIN takes a long time.^19^ Persistent HPV infection is inevitable in CIN2 or worse lesions, and with the development of microdissection techniques for target lesions and the wide application of high‐sensitive HPV genotyping methods, it is now feasible to accurately detect HPV in tissue specimens.

A previous study from our group showed that at least one HR‐HPV or pHR‐HPV can be detected in CIN2 or CIN3 FFPE tissues,^37^ which is consistent with the present results (HPV‐positive rate was 100% in CIN2 tissues). One case (case no. 71) was finally diagnosed as CIN2, after a negative result in the biopsy tissue and a positive result in the postoperative (LEEP) tissue. Tracing back to this case (case no. 71), we found that the patient’s condition was complicated by severe cervical inflammation, therefore, the false‐negative result of the HPV test in the biopsy tissue may be caused by overlooking the real lesion of the cervix or owing to severe inflammation with necrosis, leading to inadequate tissue in the specimen, insufficient specimen collected, or poor DNA extraction and quality.

Among the 10 CIN2 cases, HPV16 was the most common type, followed by HPV51/52, and nine of them were single‐type infections. The exception was case no. 68, which was positive for both HPV42 and HPV51 (Table 4). After reviewing the HE‐stained section of this case, we found that CIN2 and CIN1 coexist, however, we could not determine the specific responsible HPV type for each lesion.

There are 27 cases with only LR‐HPV infection in cervical biopsy tissue, which accounts for 43.5% of the 62 CIN1 patients. Interestingly, all of them were diagnosed with CIN1 even after LEEP or conization. This means that if the value of the case with only LR‐HPV infection is used as the cutoff value, the sensitivity of prediction for CIN2+ is 100%, and the negative predictive value of coexistence with CIN2 is also 100%. However, changing the cutoff value to any HPV‐type infection results in a negative predictive value of 94.1% and a sensitivity of 90.0% as one CIN2 case (case no. 71) tested negative in the HPV test as mentioned above. Therefore, the evidence for this is still insufficient, and the limitation of the present research is the small size of the samples. A total of 72 cases were eligible, and only 10 cases were pathological upgrade after LEEP or conization. Multicenter with large sample size study is needed for further research.

For case no. 13, we found HPV16, HPV52 in biopsy tissue, while only HPV16 was found in cervical canal cells. This may be due to the uniplex E6/E7 PCR HPV detection method has higher sensitivity, and the HPV52 virus load in cervical canal cells is too low to be detected. For the other three cases, case nos. 24, 38, and 41, we think the HPV genotypes detected in cervical canal cells may not be the types that eventually lead to lesions, they may only be temporary infection, and the responsible HPV that really causes lesions in cervical canal cells may not be detected. This difference may be caused by different sensitivities between the two HPV detection methods, which is also one of the limitations of this study.

In conclusion, the strength of this study is that we detected responsible HPV genotype in the low‐grade SIL lesions by the methods of manual microdissection with FFPE tissue specimens and the E6/E7 uniplex PCR. In the present study, we believe that patients with cervical biopsy tissue diagnosed with CIN1 and with a negative or only low‐risk HPV type result can be considered for follow‐up without further treatment such as LEEP or conization. Conversely, in cases of cervical biopsy tissue diagnosed with CIN1 positive for high‐risk HPV (especially HPV16, 18, 51, 52, and 58), surgery (LEEP or conization), or a close follow‐up program can be selected, though this warrants further investigation in multicenter trials with large samples.

## CONFLICT OF INTEREST

There is no conflict of interest in the paper.

## AUTHORS CONTRIBUTIONS

Conceptualization, H. Wang and S. Zhang; Methodology, H. Wang; Statistical analysis, X. Han; Pathological analysis, Z. He; Data curation, D. Zhang; Writing—original draft preparation, H. Wang; Writing—review and editing, S. Zhang; Visualization, S. Zhang; Supervision, S. Zhang; Project administration, S. Zhang. All authors have read and agreed to the published version of the manuscript.

## ETHICAL STATEMENT

Consent was obtained from all subjects, and the protocol was approved by the Ethics Committee of China Medical University Shengjing Hospital.

## Data Availability

Data sharing is not applicable to this article as no new data were created or analyzed in this study.
